# Association between sarcopenic obesity and cardiovascular diseases: the role of systemic inflammation indices

**DOI:** 10.3389/fmed.2025.1581146

**Published:** 2025-06-23

**Authors:** Yuhong Luo, Chen Xin, Yuhua Liu, Yan Xu, Guixin Liu, Binru Han

**Affiliations:** 1 School of Nursing, Capital Medical University, Beijing, China; 2 Xuanwu Hospital, Capital Medical University, Beijing, China

**Keywords:** sarcopenic obesity, cardiovascular diseases, inflammation, systemic inflammation response index SIRI, aggregate index of systemic inflammation, AISI

## Abstract

**Background:**

Research on the relationship between sarcopenic obesity (SO) and multiple cardiovascular diseases is limited, and the regulatory roles of the Aggregate Index of Systemic Inflammation (AISI) and Systemic Inflammation Response Index (SIRI) remain underexplored.

**Methods:**

This retrospective observational study included participants aged ≥50 years who underwent routine health examinations between January 1 and December 31, 2024. SO was defined as a low skeletal muscle mass-to-weight ratio (< 33.9% in females, <39.3% in males) and high visceral fat area (≥100 cm^2^). Participants were categorized into four groups: normal, sarcopenia, obesity, and SO. Adjusted multivariate analysis examined the association between SO and cardiovascular multimorbidities. The moderating effects of AISI and SIRI were analyzed using the Johnson–Neyman method and SPSS Process Macro.

**Results:**

This cross-sectional study included 1,010 participants aged ≥50 years. SO was significantly associated with endothelial dysfunction, arterial stiffness, degenerative heart valve disease, and carotid atherosclerosis, with odds ratios (95% confidence intervals) of 1.575 (1.017, 2.441), 1.382 (1.050, 1.818), 1.664 (1.033, 2.681), and 1.430 (1.022, 2.001), respectively. The Johnson–Neyman test identified AISI = 133.48 and SIRI = 0.58 as critical points for significant associations.

**Conclusion:**

SO is independently associated with increased cardiovascular disease risk. AISI and SIRI serve as biomarkers for risk stratification, highlighting the need for targeted management to improve cardiovascular outcomes in patients with SO.

## 1 Introduction

Sarcopenic obesity (SO), a metabolic syndrome characterized by the coexistence of reduced muscle mass and increased body fat, is increasingly prevalent among older adults (1). Epidemiological studies estimate the global prevalence of SO in individuals aged ≥65 years at 10–15%, rising to 20–30% among older adults with obesity (2). SO is not merely a combination of sarcopenia and obesity but a complex pathological condition strongly linked to chronic diseases, including cardiovascular diseases (CVDs), type 2 diabetes, and metabolic syndrome (3). For instance, a longitudinal study found that patients with SO have a 1.5–2 times higher risk of type 2 diabetes than do those with either obesity or sarcopenia alone (4). As global aging accelerates, the rising prevalence of SO presents a major public health challenge.

CVDs remain the leading cause of mortality and disability worldwide. According to the World Health Organization (5), approximately 18 million deaths annually are attributed to CVDs, accounting for 31% of global mortality. Among these, endothelial dysfunction, arterial stiffness, degenerative valvular heart disease, and carotid atherosclerosis are highly prevalent and severe cardiovascular conditions (6). The pathophysiology of these diseases involves endothelial cell damage, chronic inflammation, oxidative stress, and lipid metabolism disorders, leading to structural and functional vascular abnormalities (7). For example, arterial stiffness affects >60–70% of individuals aged ≥65 years, while carotid atherosclerosis occurs in >50% of this population (8). The growing burden of these diseases in aging societies strains healthcare systems.

Emerging evidence suggests a strong association between SO and CVDs. SO may promote CVD progression through chronic inflammation, insulin resistance, oxidative stress, and metabolic dysregulation (2). For instance, adipose tissue in patients with SO secretes excessive proinflammatory cytokines, such as tumor necrosis factor-alpha (TNF-*α*) and interleukin-6 (IL-6), contributing to endothelial dysfunction and arterial stiffness (9). Additionally, reduced muscle mass among patients with SO may lower metabolic rates, exacerbating obesity and cardiovascular risks (10).

Inflammation plays a central role in SO and CVD pathogenesis. Chronic low-grade inflammation, a hallmark of SO, activates inflammatory signaling pathways that promote endothelial injury and atherosclerosis (11). Recently, novel inflammatory biomarkers, such as the Aggregate Index of Systemic Inflammation (AISI) and the Systemic Inflammation Response Index (SIRI), have been widely adopted to assess systemic inflammatory status (12). These indices provide a more comprehensive measure of inflammation, offering new insights into the relationship between SO and CVDs.

Despite studies linking SO to individual cardiovascular conditions, comprehensive research on its association with multiple CVDs remains scarce. Additionally, the regulatory effects of AISI and SIRI in this relationship have not been thoroughly explored. Therefore, this study aimed to investigate the association between SO and four cardiovascular conditions—endothelial dysfunction, arterial stiffness, degenerative valvular heart disease, and carotid atherosclerosis—and to analyze the mediating roles of AISI and SIRI in these relationships further.

## 2 Materials and methods

### 2.1 Study design and participants

This study is a retrospective observational study. It included participants aged ≥50 years who underwent routine health examinations at Xuanwu Hospital, Capital Medical University, between January 1 and December 31, 2024. All eligible individuals who met the inclusion and exclusion criteria during this one-year period were consecutively included in the study. Therefore, no *a priori* sample size calculation was performed. However, the final sample size was deemed sufficient for statistical analysis based on the number of variables and observed effect sizes. Exclusion criteria included acute or chronic infections, malignancies, congenital heart disease, severe liver or kidney disease, infectious or autoimmune diseases; the presence of metal stents, pacemakers, or other devices interfering with body composition measurements; the use of weight-loss medications or drugs affecting body weight (e.g., laxatives) within the past 6 months; and incomplete physical examinations or medical records. This study was approved by the Ethics Committee of Xuanwu Hospital, Capital Medical University (Approval No. KS2024309) and conducted in accordance with the principles outlined in the Declaration of Helsinki. As the study involved no direct interaction with participants and relied solely on anonymized data, the Ethics Committee granted a waiver of informed consent.

### 2.2 Measurement of laboratory indicators and SIRI and AISI calculation

After an 8-h fast, laboratory indicators were collected in the morning. A fully automated electrochemical analyzer was used to measure the neutrophil, lymphocyte, monocyte, and platelet counts. Fasting blood glucose level was assessed using the glucose oxidase method. A fully automated biochemical analyzer was used to measure fasting triglycerides (TG), total cholesterol (TC), high-density lipoprotein cholesterol (HDL-C), low-density lipoprotein cholesterol (LDL-C), creatinine, and uric acid levels. SIRI and AISI were calculated as follows: SIRI = neutrophil count × monocyte count / lymphocyte count; AISI = neutrophil count × monocyte count × platelet count / lymphocyte count.

### 2.3 Anthropometric measurements and bioimpedance analysis (BIA)

Body weight and height were measured using a digital scale, and body mass index (BMI) was calculated as weight (kg) divided by the squared value of height (m^2^). A well-trained examiner measured waist circumference using a measuring tape at the midpoint between the lower costal margin and anterior superior iliac crest. Blood pressure was measured on the right brachial artery using a calibrated electronic sphygmomanometer. Smoking, tea, or coffee consumption was prohibited 30 min before measurement. Participants rested in a seated position with their back supported for 15 min before the measurement. Blood pressure was recorded three times at 1–2-min intervals, and the average was used.

Body composition was assessed using BIA with an InBody 720 Body Composition Analyzer (InBody Co., Ltd., Seoul, Korea). Participants stood for 5–10 min with legs slightly apart and arms slightly abducted before grasping the analyzer handles to ensure proper electrode contact. The required body composition parameters, including percent body fat, visceral fat area (VFA), and skeletal muscle mass (SMM), were obtained.

### 2.4 Definition of sarcopenia, obesity and SO

Based on the study by Janssen et al., participants were classified as normal if their muscle percentage was within ≥1 standard deviation of the sex-specific mean for young adults (aged 18–39 years) (13, 14); participants who did not meet the definition of normal muscle percentage were considered to have sarcopenia. In this study, the muscle percentage (mean ± standard deviation) was 42.28 ± 2.95% for young males and 36.71 ± 2.72% for young females. Therefore, sarcopenia was defined as a muscle percentage of 39.3% for males and 33.9% for females. Obesity was defined according to the Chinese Medical Association (15) criteria (VFA ≥ 100 cm^2^). SO was defined by the presence of both low skeletal muscle mass-to-weight ratio (<33.9% in females and <39.3% in males) and high VFA (≥100 cm^2^).

### 2.5 Brachial-ankle pulse wave velocity (baPWV) measurement

The arterial stiffness in this study was assessed using brachial-ankle pulse wave velocity (baPWV), which is a widely recognized and non-invasive method. The baPWV was measured using an automatic device (BP-203RPEIII; Omron Healthcare, China) in a temperature-controlled room (22–25°C) by trained healthcare professionals. Participants rested for >5 min before the test and were instructed not to smoke beforehand. During the test, participants lay supine with palms facing up. Blood pressure cuffs were applied to the upper arms and lower legs, and an ECG sensor was placed over the chest. ECG collection devices were attached to both wrists. Two measurements were taken, and the second was used as the final result. According to the Chinese Expert Consensus on Clinical Application of Synchronous Measurement of Four-Limb Blood Pressure and Ankle-Brachial Pulse Wave Velocity (2020), baPWV values were categorized as normal (<1,400 cm/s), borderline (1,400–1,800 cm/s), or abnormal (>1,800 cm/s) (16). Therefore, we adopted baPWV >1,800 cm/s as the cutoff point to define increased arterial stiffness, consistent with previous studies and clinical recommendations.

### 2.6 Assessment of endothelial function

Endothelial function was assessed using flow-mediated dilation (FMD). Participants rested supine for 20 min before measurement. A 7.5–12.0 MHz ultrasound diagnostic device (Mindray M9, Mindray, China) was used to measure the right brachial artery diameter (D0) 2–5 cm above the elbow. A cuff placed on the forearm (2–3 cm below the elbow) was inflated to 200–250 mmHg for 5 min and then deflated. Within 1 min after cuff deflation, the brachial artery diameter was measured during the period of peak vasodilation. Specifically, the diameter was recorded across three consecutive cardiac cycles, and the average of these measurements was taken as D1. FMD (%) was calculated as (D1 − D0) / D0 × 100%. An FMD value less than 10% was considered indicative of endothelial dysfunction (17).

### 2.7 Carotid atherosclerosis measurements

Carotid artery examination was performed using a B-mode ultrasound imaging device (Mindray M9, Mindray, China) with a 7.5–10.0 MHz probe by a qualified sonographer. Participants lay in a supine position with the neck extended and head slightly elevated and tilted toward the opposite side. Carotid intima-media thickness (CIMT) was measured on both sides of the common carotid artery 1 cm below the bifurcation at the posterior wall, and the final CIMT value was the average of value of both sides. The presence of carotid plaque was defined as meeting any one of the following criteria: (1) CIMT ≥1.5 mm; (2) protrusion of atherosclerosis into the lumen of the artery with ≥50% thickness compared to that of the surrounding area; and (3) presence of distinct areas of hyperechogenicity (18).

### 2.8 Degenerative heart valve disease (DHVD)

The conventional two-dimensional and color Doppler echocardiographic examination was performed using an ultrasound echocardiography device (GE Vivid E9; GE, USA). Participants were examined in a supine or slightly left lateral position. Scanning was conducted on the following planes: parasternal short axis of the aorta, left ventricular long axis, apical long axis of the left ventricle, and apical four-chamber view. The diagnostic criteria for DHVD were as follows: (1) presence of focal or mass-like calcification or thickening of the aortic, mitral, tricuspid, or pulmonary valves or their annuli, with a thickening >3 mm and enhanced echogenicity; (2) functional examination showing restricted valve closure, accompanied by signs of regurgitation and insufficiency, or observation of valve stenosis (19).

### 2.9 Statistical analysis

Categorical variables are presented as frequencies and percentages. Continuous variables are expressed as means ± standard deviations for normally distributed data or as medians (Q1, Q3) for non-normally distributed data. Group comparisons were performed using ANOVA, the Kruskal–Wallis H test, or Pearson’s chi-square test, as appropriate. Logistic regression was used to assess associations between variables. To explore whether inflammatory indices (e.g., AISI and SIRI) moderated the relationship between SO and CVDs, moderation analysis was performed using Model 1 of the PROCESS macro (version 4.1) in SPSS. The Johnson–Neyman (J-N) technique was applied to identify regions of significance across moderator values. This approach, commonly used in clinical and public health research, allows precise identification of the threshold at which the moderating effect becomes statistically significant (20, 21). Statistical analyses were performed using SPSS (version 26.1). *p* values <0.05 were considered statistically significant.

## 3 Results

### 3.1 Baseline characteristics based on the SO phenotype

**Figure 1:**
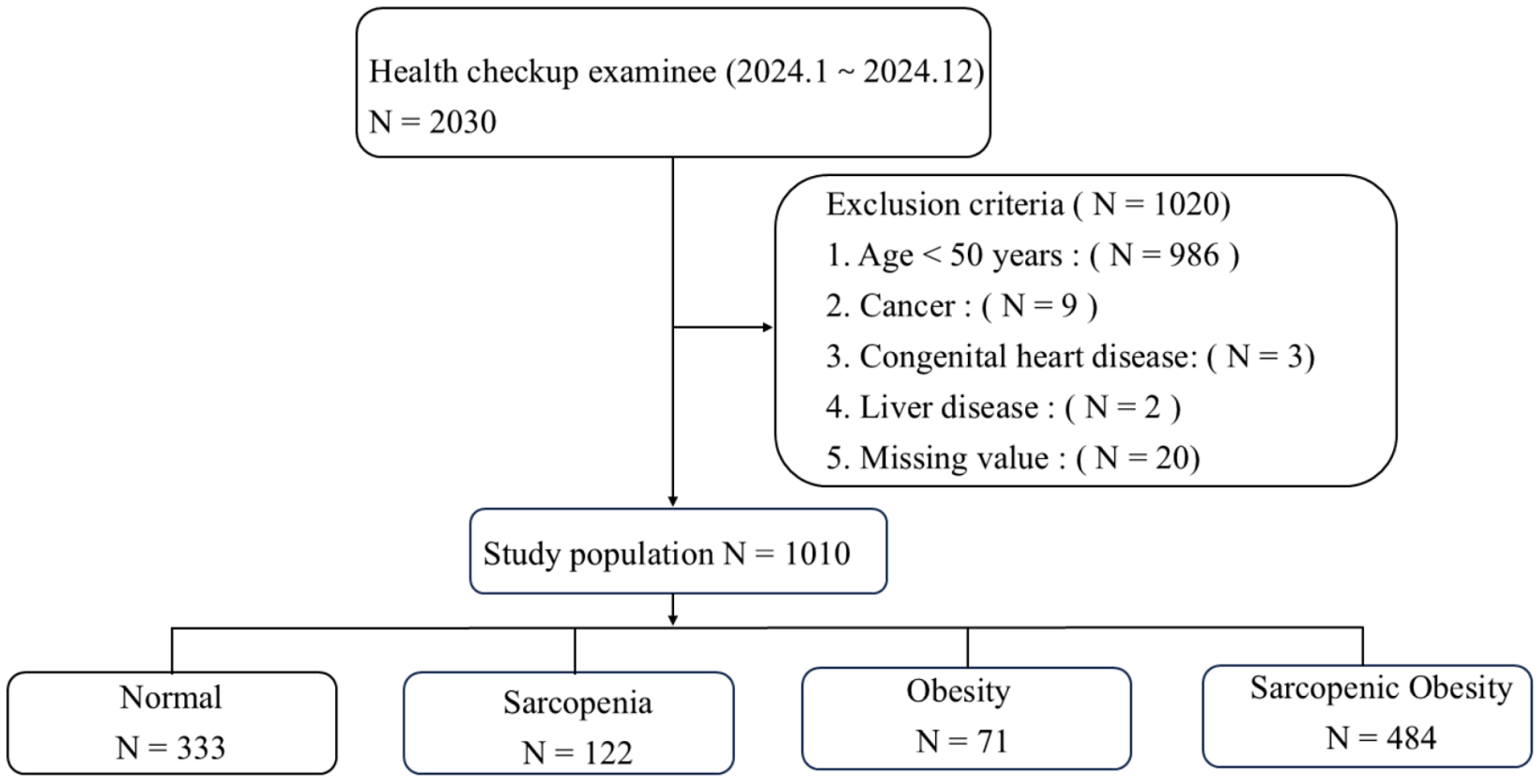
Participant flow chart.

We analyzed data from 2,030 health checkup examinees and excluded 1,020 individuals based on the exclusion criteria, resulting in a final sample of 1,010 participants (Figure 1). The baseline characteristics are summarized in Table 1. Among the participants, 333 (32.97%) had non-sarcopenic non-obese status, 122 (12.08%) had sarcopenic non-obese status, 71 (7.03%) had non-sarcopenic obesity, and 484 (47.92%) had SO. The mean age was significantly higher in the SO group. Additionally, the prevalence of arterial stiffness, endothelial dysfunction, DHVD, carotid atherosclerosis, diabetes mellitus, hypertension, and non-alcoholic fatty liver disease was significantly higher in the SO group than in the other groups (*p* < 0.05).

**Table 1:** Baseline characteristics based on the sarcopenic obesity phenotype.

Characteristic	Total(*n* = 1,010)	Normal (*n* = 333)	Sarcopenia (*n* = 122)	Obesity (*n* = 71)	Sarcopenic Obesity (*n* = 484)	*p*-value
Sex						*p* < 0.001
Male	514 (50.89%)	197 (59.16%)	47 (38.52%)	61 (85.91%)	209 (43.18%)	
Female	496 (49.11%)	136 (40.84%)	75 (61.48%)	10 (14.08%)	275 (56.83%)	
Age (y)						
	60.30 ± 7.79	58.75 ± 7.26	57.49 ± 5.59	64.21 ± 7.73	61.51 ± 8.17	*p* < 0.001
Cardiovascular diseases						
Arterial stiffness	372 (36.87%)	98 (29.43%)	41 (33.61%)	29 (41.43%)	204 (42.15%)	*p* = 0.002
Endothelial dysfunction	101 (10.00%)	24 (7.21%)	11 (9.02%)	7 (10.00%)	59 (12.19%)	*p* = 0.133
DHVD	89 (8.82%)	21 (6.31%)	6 (4.92%)	9 (12.86%)	53 (10.95%)	*p* = 0.028
Carotid atherosclerosis	193 (19.13%)	49 (14.71%)	24 (19.67%)	11 (15.71%)	109 (22.52%)	*P* = 0.039
Comorbidity						
Diabetes mellitus	134 (13.28%)	29 (8.70%)	21 (17.21%)	8 (11.43%)	76 (15.70%)	*p* = 0.016
Hypertension	366 (36.27%)	76 (22.82%)	44 (36.07%)	22 (31.43%)	224 (46.28%)	*p* < 0.001
NAFLD	430 (42.62%)	67 (20.12%)	63 (51.64%)	21 (30.00%)	279 (57.64%)	*p* < 0.001
Anthropometric data						
Height (cm)	162.70 ± 9.28	164.33 ± 9.98	165.98 ± 8.46	162.89 ± 7.33	160.71 ± 8.782	*p* < 0.001
Weight (kg)	68.33 ± 12.12	63.35 ± 10.29	73.02 ± 11.53	64.05 ± 10.36	71.20 ± 12.32	*p* < 0.001
BMI (kg/m^2^)	25.92 ± 7.62	23.90 ± 12.32	26.35 ± 2.35	24.54 ± 2.63	27.41 ± 3.02	*p* < 0.001
WC (cm)	87.39 ± 10.08	81.92 ± 8.71	88.98 ± 9.26	86.01 ± 6.46	90.95 ± 9.91	*p* < 0.001
Body fat mass	22.79 ± 6.33	16.83 ± 3.40	23.52 ± 2.17	19.48 ± 3.09	27.19 ± 5.25	*p* < 0.001
SMM	24.88 ± 5.58	25.54 ± 5.55	27.24 ± 6.30	24.68 ± 3.83	23.86 ± 54.39	*p* < 0.001
VFA (cm^2^)	114.33 ± 37.40	76.98 ± 15.98	115.01 ± 11.07	90.02 ± 9.64	143.37 ± 28.41	*p* < 0.001
SMM/WT (%)	36.27 ± 4.34	40.03 ± 3.61	36.91 ± 2.86	37.70 ± 1.75	33.32 ± 2.99	*p* < 0.001
SBP (mmHg)	132.90 ± 18.32	126.58 ± 15.06	134.32 ± 18.61	130.53 ± 17.02	137.23 ± 19.19	*p* < 0.001
DBP (mmHg)	79.31 ± 11.10	76.94 ± 10.08	80.48 ± 12.14	79.03 ± 10.08	80.69 ± 11.40	*p* < 0.001
Biochemical markers						
Glucose (mg/dL)	6.35 ± 14.97	5.62 ± 1.52	5.98 ± 1.59	5.88 ± 1.61	7.02 ± 1.54	0.596
Total cholesterol (mg/dL)	5.23 ± 1.90	5.17 ± 1.23	5.29 ± 0.96	4.85 ± 0.97	5.32 ± 2.46	0.234
LDL-C (mg/dL)	3.17 ± 0.98	3.15 ± 1.03	3.24 ± 0.89	2.93 ± 0.78	3.21 ± 0.99	0.127
HDL-C (mg/dL)	1.37 ± 0.38	1.47 ± 0.38	1.30 ± 0.30	1.31 ± 0.34	1.33 ± 0.39	*p* < 0.001
Triglyceride (mg/dL)	1.86 ± 2.24	1.61 ± 2.19	2.05 ± 1.82	2.09 ± 4.10	1.96 ± 1.97	0.078
Creatinine (mg/dL)	63.38 ± 15.51	65.29 ± 15.29	62.29 ± 12.95	70.79 ± 13.54	61.27 ± 16.05	*p* < 0.001
Blood cell count						
WBC/(10^9 /L)	6.07 ± 1.55	5.81 ± 1.53	5.85 ± 1.43	6.09 ± 1.49	6.29 ± 1.57	*p* < 0.001
NEUT /(10^9 /L)	3.54 ± 1.22	3.38 ± 1.16	3.35 ± 1.10	3.63 ± 1.14	3.69 ± 1.28	*p* = 0.001
	6.78 ± 2.38	6.80 ± 2.29	7.10 ± 2.23	5.88 ± 1.86	6.80 ± 2.52	*p* = 0.007
MONO/(10^9 /L)	0.32 ± 0.11	0.31 ± 0.11	0.30 ± 0.09	0.35 ± 0.13	0.33 ± 0.11	*p* < 0.001
PLT /(10^9 /L)	220.83 ± 54.85	217.08 ± 52.82	222.15 ± 44.12	206.69 ± 45.81	225.13 ± 59.28	*p* = 0.026
Blood Cell Inflammatory Index						
AISI	111.53 (74.43, 164.13)	106.54 (67.28, 153.88)	113.23 (86.50, 182.42)	100.06 (72.19, 144.58)	116.47 (78.13, 171.73)	*p* = 0.004
SIRI	0.52 (0.37, 0.73)	0.49 (0.34, 0.69)	0.47 (0.33, 0.65)	0.46 (0.34, 0.65)	0.54 (0.37, 0.74)	*p* < 0.001

DHVD, degenerative heart valve disease; NAFLD, nonalcoholic fatty liver disease; BMI, body mass index; SMM, skeletal muscle mass; VFA, visceral fat area; SMM/WT, skeletal muscle mass/weight; WC, waist circumference; SBP, systolic blood pressure; DBP, diastolic blood pressure; LDL-C, low-density lipoprotein-cholesterol; HDL-C, high-density lipoprotein-cholesterol; WBC, white blood cells; NEUT, neutrophils; LYM, lymphocytes; MONO, monocytes; PLT, platelets; AISI, Aggregate Index of Systemic Inflammation; SIRI, Systemic Inflammatory Response Index.

Significant differences were observed in white blood cell, neutrophil, lymphocyte, monocyte, and platelet counts among the four subgroups (*p* < 0.001). Both SIRI and AISI were significantly higher in the SO group than in the other groups (*p* < 0.001 and *p* = 0.004, respectively), indicating a more severe inflammatory state in the SO group (Table 1).

### 3.2 Odds ratios (ORs) for CVDs based on the SO phenotype

We performed multivariable logistic regression analysis, adjusting for factors including sex, age, hypertension, diabetes, TG, TC, LDL, and creatinine. After adjustment, the SO group showed significant associations with endothelial dysfunction, arterial stiffness, DHVD, and carotid atherosclerosis. The ORs (95% confidence intervals) were 1.575 (1.017, 2.441), 1.382 (1.050, 1.818), 1.664 (1.033, 2.681), and 1.430 (1.022, 2.001), respectively (Table 2).

**Table 2:** Comparison of odds ratios for cardiovascular diseases across sarcopenic obesity phenotypes.

Outcome	Variable	Model 1	Model 2	Model 3
		OR (95%CI)	OR (95%CI)	OR (95%CI)
Endothelial dysfunction	Normal	reference	reference	reference
	SO	1.703 (1.111, 2.610)^*^	1.592 (1.030, 2.463)^*^	1.575 (1.017, 2.441)^*^
	Sarcopenia	0.910 (0.397, 2.089)	0.978 (0.424, 2.256)	0.966 (0.415, 2.245)
	Obesity	0.903 (0.464, 1.757)	0.865 (0.443, 1.688)	0.859 (0.439, 1.678)
Arterial stiffness	Normal	reference	reference	reference
	SO	1.519 (1.167, 1.976)^**^	1.370 (1.046, 1.794)^*^	1.382 (1.050, 1.818)^*^
	Sarcopenia	1.240 (0.745, 2.063)	1.385 (0.826, 2.323)	1.375 (0.819, 2.308)
	Obesity	0.863 (0.575, 1.293)	0.813 (0.539, 1.226)	0.808 (0.535, 1.219)
DHVD	Normal	reference	reference	reference
	SO	0.650 (0.412, 1.027)	1.517 (0.952, 2.418)	1.664 (1.033, 2.681)^*^
	Sarcopenia	1.165 (0.543, 2.499)	1.148 (0.531, 2.481)	1.140 (0.527, 2.464)
	Obesity	0.647 (0.272, 1.538)	0.642 (0.270, 1.528)	0.663 (0.278, 1.586)
Carotid atherosclerosis	Normal	reference	reference	reference
	SO	1.490 (1.077, 2.063)^*^	1.439 (1.030, 2.011)^*^	1.430 (1.022, 2.001)^*^
	Sarcopenia	0.622 (0.315, 1.230)	0.657 (0.331, 1.304)	0.652 (0.328, 1.298)
	Obesity	1.198 (0.736, 1.949)	1.158 (0.709, 1.890)	1.164 (0.712, 1.903)

*P < 0.05; **p < 0.01; Values are presented as odds ratios (95% confidence interval). OR, odds ratio; CI, confidence interval; DHVD, degenerative heart valve disease; SO, sarcopenic obesity. Model 1: adjusted for age and sex; Model 2: adjusted for age, sex, hypertension, and diabetes; Model 3: adjusted for sex, age, hypertension, diabetes, triglycerides, total cholesterol, low-density lipoprotein, and creatinine. *p*-values were calculated using the logistic regression analysis.

### 3.3 Simple slope analysis and J-N analysis of the AISI and SIRI moderation effects

**Figure 2:**
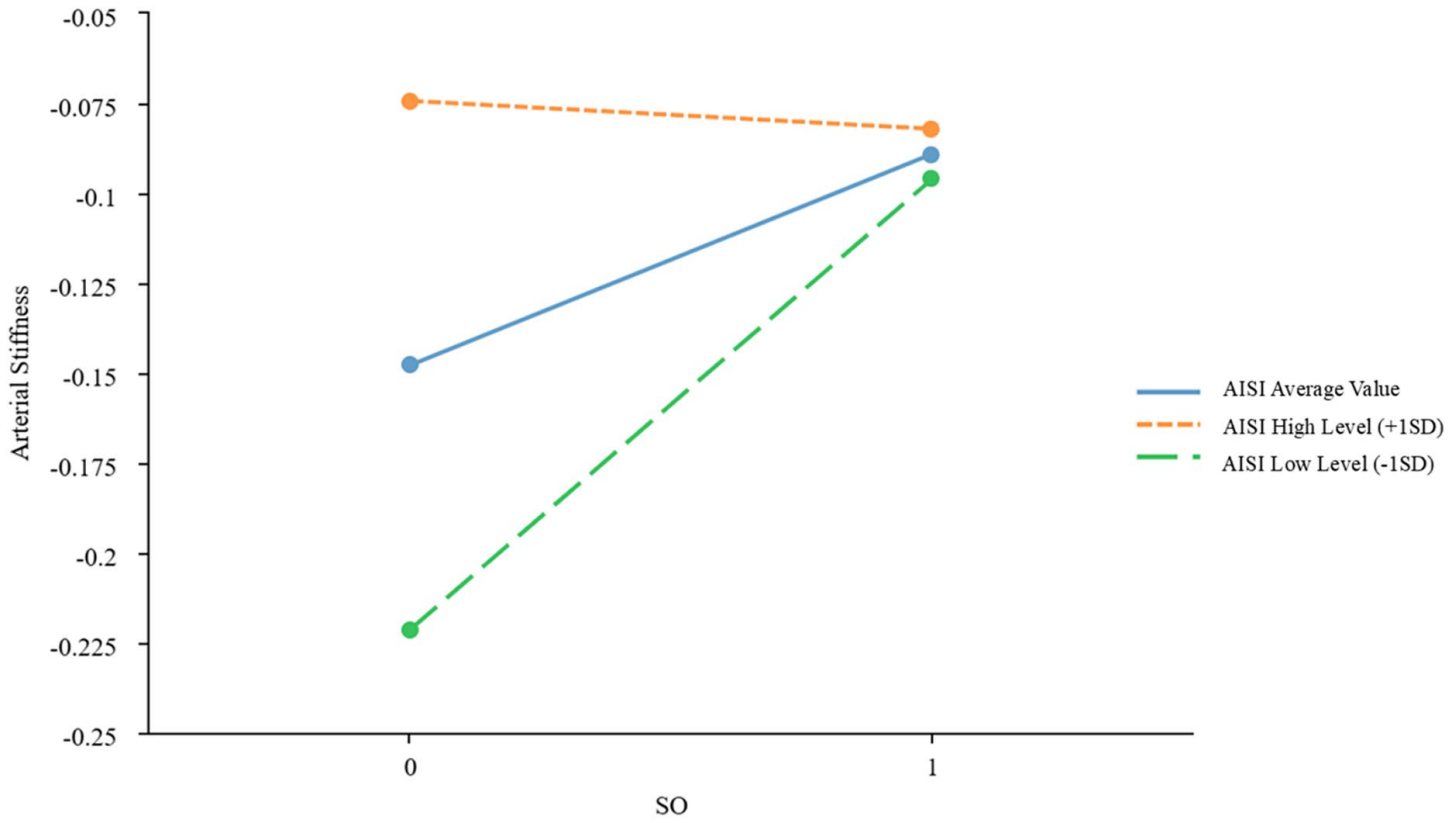
Simple slope plot of the moderating effect of AISI AISI, Aggregate Index of Systemic Inflammation; SD, standard deviation.

**Figure 3:**
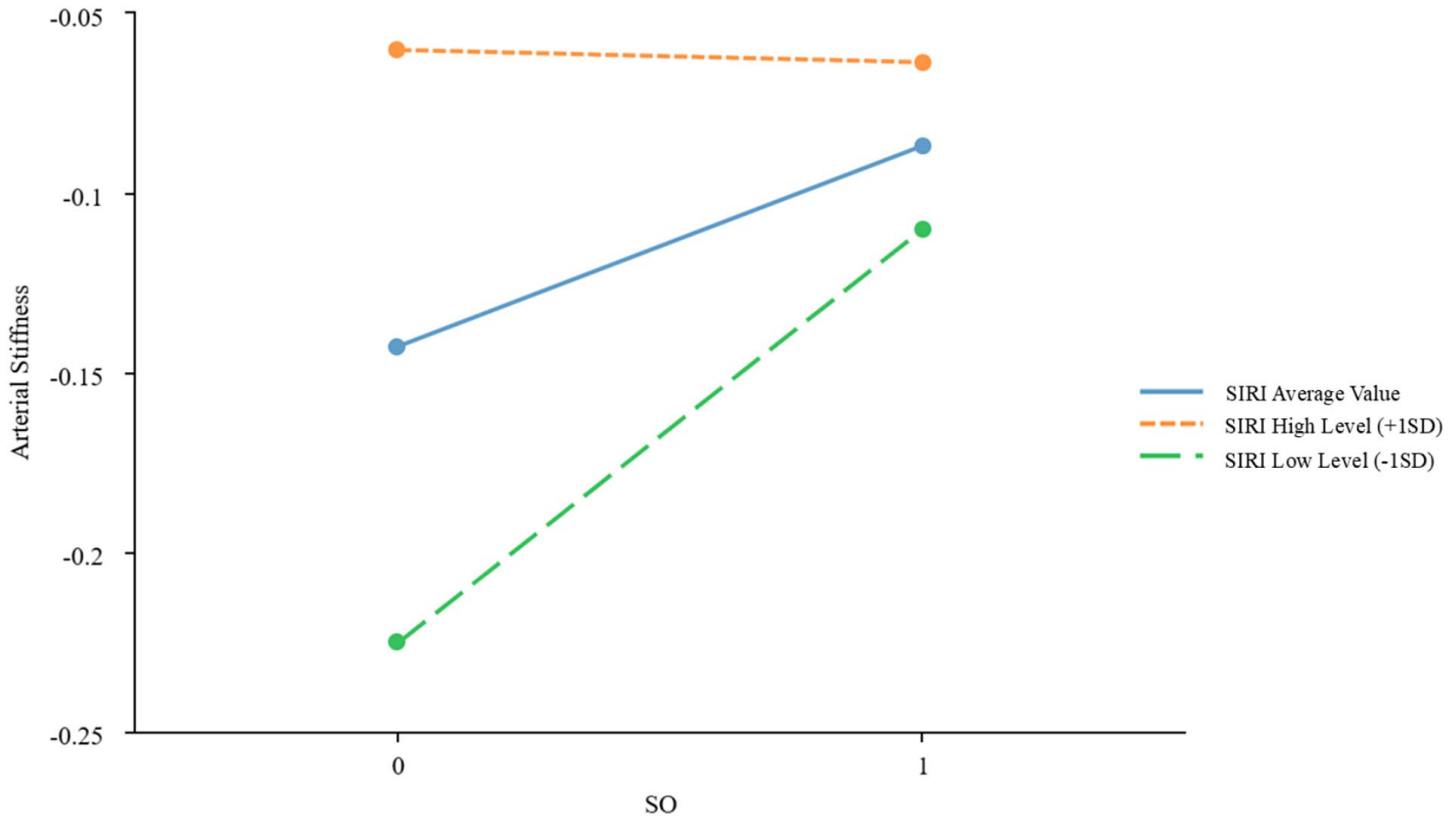
Simple slope plot of the moderating effect of SIRI. SIRI, Systemic Inflammatory Response Index; SD, standard deviation.

To further examine the moderating effects of AISI and SIRI on the relationship between SO and CVDs, we conducted a simple slope analysis using the mean ± standard deviation of AISI and SIRI as criteria for high and low levels, respectively. The results indicated significant moderating effects of AISI and SIRI on the relationship between SO and atherosclerosis (*β* = −0.067, *p* = 0.022; *β* = −0.059, *p* = 0.039, respectively), as shown in Figures 2, 3 and Table 3. However, AISI and SIRI did not significantly moderate the relationship between SO and endothelial dysfunction, DHVD, or carotid atherosclerosis (Supplementary Tables S1–S3).

**Table 3:** Moderating effect of AISI and SIRI on the relationship between SO and arterial stiffness.

Variables	AISI					SIRI			
	*β*	SE	*t*	*P*-value		*β*	SE	*t*	*P*-value
Constant	−0.148	0.156	−0.946	0.345	Constant	−0.143	0.156	−0.915	0.360
Age	−0.001	0.002	−0.498	0.618	Age	−0.001	0.002	−0.685	0.494
Sex	0.020	0.031	0.639	0.523	Sex	0.033	0.031	1.037	0.300
SBP	0.004	0.001	4.777	0.000^**^	SBP	0.004	0.001	4.818	0.000^**^
Triglycerides	0.005	0.007	0.749	0.454	Triglycerides	0.005	0.007	0.762	0.446
Total cholesterol	0.010	0.010	1.012	0.312	Total cholesterol	0.010	0.010	1.039	0.299
LDL-C	−0.024	0.018	−1.312	0.190	LDL	−0.021	0.018	−1.179	0.239
Glucose	0.001	0.001	1.305	0.192	Glucose	0.001	0.001	1.216	0.224
**SO**	0.065	0.031	2.068	0.039*	SO	0.065	0.031	2.068	0.039*
**AISI**	0.073	0.021	3.545	0.000**	SIRI	0.083	0.021	3.901	0.000**
AISI*SO	−0.067	0.030	−2.213	0.022*	SIRI*SO	−0.059	0.030	−1.973	0.039*

*P < 0.05; **P < 0.01; SBP, systolic blood pressure; LDL-C, low-density lipoprotein cholesterol; SO, sarcopenic obesity; AISI, Aggregate Index of Systemic Inflammation; SIRI, Systemic Inflammatory Response Index.

The J-N test identified AISI = 133.48 as the critical threshold for significance. When AISI was below this value, the effect of SO on atherosclerosis was significant; above this value, the effect gradually diminished. For SIRI, 0.58 marked the point where the effect of SO on atherosclerosis became significant. Below this value, the effect was stronger, while above it, the effect weakened.

## 4 Discussion

This study highlights significant associations between SO and multiple CVDs, including arterial stiffness, endothelial dysfunction, DHVD, and carotid atherosclerosis. Our findings align with those of prior research (22–24) while extending current knowledge by providing novel insights into the mediating role of systemic inflammation, as measured via SIRI and AISI, in these relationships.

Our analysis demonstrated that SO was independently associated with a higher prevalence of arterial stiffness, endothelial dysfunction, DHVD, and carotid atherosclerosis, even after adjusting for confounders such as age, sex, hypertension, diabetes, and lipid profiles. This contrasts with isolated sarcopenia or obesity, which show weaker CVD correlations. For instance, an 8-year community-based cohort study of 3,366 older adults without baseline CVD found that SO increased CVD risk by 23%, whereas sarcopenia or obesity alone did not significantly elevate the risk (25). In another study, Farmer et al. (26) investigated the association of sarcopenia and obesity with CVD and reported that obesity alone, sarcopenia alone, and SO were all associated with increased cardiovascular and all-cause mortality, as well as cardiovascular events. These findings reinforce the association between SO and CVD.

Patients with SO may exhibit endothelial dysfunction due to chronic inflammation and oxidative stress (9). Our results confirm these observations. Bak et al. (27) further identified that the risk of high baPWV (>1,800 cm/s) in the SO group was 2.40 times higher than that in the non-SO obese group, a finding corroborated by our study. Notably, previous research has primarily examined the relationship between SO and atherosclerosis, whereas our study suggests that SO may also contribute to degenerative changes in cardiac valves through chronic inflammation and metabolic dysregulation (28, 29). This is supported by the significantly higher prevalence of DHVD in the SO group than that for other phenotypes. Moreover, the association between SO and carotid atherosclerosis further reinforces its systemic cardiovascular risk, aligning with the findings of Motoya et al., who reported a strong correlation between SO, defined by VFA, and carotid atherosclerosis (30). However, Donini et al. (31) found a weaker association between SO and carotid atherosclerosis, suggesting that population heterogeneity (e.g., race and age), differences in SO diagnostic criteria (muscle strength and mass assessment methods), and atherosclerosis detection methods (e.g., ultrasound plaque assessment and baPWV) may contribute to these discrepancies. Future studies should conduct multicenter cohort research using standardized phenotype definitions, such as the EWGSOP2 consensus criteria, and methodological approaches to enhance comparability.

SO may promote CVD through multiple mechanisms. Visceral adipose tissue increases circulating free fatty acids, contributing to endothelial injury (32). Proinflammatory cytokines, including IL-6, TNF-*α*, and leptin, coupled with reduced adiponectin levels, further impair endothelial function and arterial compliance (33, 34). Concurrently, skeletal muscle loss exacerbates insulin resistance by reducing insulin-mediated glucose uptake, a key driver of atherosclerosis (35, 36). Animal models suggest that SO upregulates NADPH oxidase activity, leading to excessive reactive oxygen species production, vascular smooth muscle cell apoptosis, and collagen deposition, which impair arterial elasticity (11).

A key finding is the markedly elevated SIRI and AISI values in patients with SO, reflecting heightened systemic inflammation. Chronic low-grade inflammation, a hallmark of SO, is implicated in cardiovascular pathogenesis (11). The J-N analysis identified threshold values (AISI = 133.48; SIRI = 0.58), below which the impact of SO on arterial stiffness was most pronounced. At higher inflammation levels, alternative mechanisms may dominate. However, SIRI and AISI did not significantly mediate endothelial dysfunction, DHVD, or carotid atherosclerosis, suggesting roles for hemodynamic or lipid-metabolic pathways in these conditions.

Our study expands upon previous research in several key areas. First, while prior studies primarily focused on the relationship between SO and individual CVDs, our research provides a comprehensive analysis across multiple cardiovascular conditions, including DHVD. Second, the inclusion of inflammatory biomarkers (SIRI and AISI) offers a more comprehensive assessment of systemic inflammation compared to that with traditional markers such as C-reactive protein or IL-6. Several limitations should be acknowledged. First, the cross-sectional design limits the ability to draw causal conclusions between SO and CVDs; longitudinal studies are necessary to further establish these associations. Second, due to the retrospective design and data constraints, we defined sarcopenia based solely on muscle mass without including muscle strength or physical performance, as recommended by international guidelines (e.g., EWGSOP2, AWGS). While this differs from consensus definitions, similar approaches have been adopted in previous studies (13, 14, 22, 37, 38), supporting the validity of our method under the given conditions. Finally, we acknowledge that the lack of assessment of dietary intake and physical activity is a limitation of this study. While this was not the primary focus of our research, it is possible that these factors may influence the relationship between SO and CVDs. Therefore, future studies should include these factors to provide a more comprehensive understanding of this association.

## 5 Conclusion

This study reveals a significant association between SO and various CVDs, highlighting that SIRI and AISI may serve as novel biomarkers for risk stratification. These findings provide a new theoretical basis for cardiovascular risk management in patients with SO. Future research should further validate these findings and explore their application in clinical practice to improve cardiovascular health outcomes in patients with SO.

## Data Availability

The original contributions presented in the study are included in the article/Supplementary material, further inquiries can be directed to the corresponding author.

## Glossary

AISI: Aggregate Index of Systemic Inflammation
ANOVA: Analysis of Variance
baPWV: Brachial–Ankle Pulse Wave Velocity
BIA: Bioelectrical Impedance Analysis
BMI: Body Mass Index
CIMT: Carotid Intima-Media Thickness
CVD: Cardiovascular Disease
DBP: Diastolic Blood Pressure
DHVD: Degenerative Heart Valve Disease
EWGSOP2: European Working Group on Sarcopenia in Older People 2
FMD: Flow-Mediated Dilation
GLP-1: Glucagon-Like Peptide-1
HDL-C: High-Density Lipoprotein Cholesterol
IL-6: Interleukin-6
J-N: Johnson–Neyman
LDL-C: Low-Density Lipoprotein Cholesterol
MONO: Monocytes
NAFLD: Non-Alcoholic Fatty Liver Disease
NEUT: Neutrophils
OR: Odds Ratio
PLT: Platelets
SBP: Systolic Blood Pressure
SGLT2: Sodium-Glucose Cotransporter-2
SIRI: Systemic Inflammation Response Index
SO: Sarcopenic Obesity
SMM: Skeletal Muscle Mass
SMM/WT: Skeletal Muscle Mass-to-Weight Ratio
TG: Triglycerides
TNF-α: Tumor Necrosis Factor-Alpha
VFA: Visceral Fat Area
WC: Waist Circumference
WBC: White Blood Cells
