# Supplementary material for: Association between sarcopenic obesity and cardiovascular diseases: the role of systemic inflammation indices
Source: Front Med (Lausanne). 2025 Jun 23;12:1581146. doi: 10.3389/fmed.2025.1581146 (PMC12230098; doi:10.3389/fmed.2025.1581146)
Supplement: Supplementary file 1 [file Table_1.docx]

**Table S1.** Moderating effect of AISI and SIRI on the relationship between SO and endothelial dysfunction

|  | AISI | | | |  | SIRI | | | |
| --- | --- | --- | --- | --- | --- | --- | --- | --- | --- |
|  | β | SE | t | p |  | β | SE | t | p |
| Constant | -0.021 | 0.099 | -0.210 | 0.834 | Constant | -0.019 | 0.099 | -0.195 | 0.846 |
| Age | -0.000 | 0.001 | -0.357 | 0.721 | Age | -0.001 | 0.001 | -0.404 | 0.687 |
| Sex | -0.029 | 0.020 | -1.466 | 0.143 | Sex | -0.027 | 0.020 | -1.353 | 0.176 |
| SBP | 0.001 | 0.001 | 1.144 | 0.253 | SBP | 0.001 | 0.001 | 1.135 | 0.257 |
| Triglycerides | 0.004 | 0.004 | 0.978 | 0.328 | Triglycerides | 0.004 | 0.004 | 1.008 | 0.314 |
| Total Cholesterol | -0.002 | 0.006 | -0.326 | 0.744 | Total Cholesterol | -0.002 | 0.006 | -0.319 | 0.750 |
| LDL-C | 0.016 | 0.012 | 1.426 | 0.154 | LDL | 0.017 | 0.012 | 1.468 | 0.142 |
| Glucose | 0.002 | 0.001 | 2.875 | 0.004^**^ | Glucose | 0.002 | 0.001 | 2.886 | 0.004^**^ |
| **SO** | 0.037 | 0.020 | 1.869 | **0.062** | **SO** | 0.037 | 0.020 | 1.860 | **0.063** |
| **AISI** | 0.004 | 0.013 | 0.284 | **0.776** | **SIRI** | 0.006 | 0.013 | 0.461 | **0.645** |
| **AISI*SO** | 0.009 | 0.019 | 0.498 | **0.619** | **SIRI*SO** | 0.005 | 0.019 | 0.285 | **0.776** |

Note: *P<0.05, **P<0.01; SBP, systolic blood pressure; LDL-C, low-density lipoprotein cholesterol; SO: sarcopenic obesity; AISI, Aggregate Index of Systemic Inflammation; SIRI, Systemic Inflammatory Response Index
