# Supplementary material for: Association between sarcopenic obesity and cardiovascular diseases: the role of systemic inflammation indices
Source: Front Med (Lausanne). 2025 Jun 23;12:1581146. doi: 10.3389/fmed.2025.1581146 (PMC12230098; doi:10.3389/fmed.2025.1581146)
Supplement: Supplementary file 2 [file Table_2.docx]

**Table S2.** Moderating effect of AISI and SIRI on the relationship between SO and DHVD

|  | AISI | | | |  | SIRI | | | |
| --- | --- | --- | --- | --- | --- | --- | --- | --- | --- |
|  | β | SE | t | p |  | β | SE | t | p |
| Constant | -0.204 | 0.093 | -2.193 | 0.029^*^ | Constant | -0.202 | 0.093 | -2.171 | 0.030^*^ |
| Age | 0.005 | 0.001 | 3.964 | 0.000^**^ | Age | 0.005 | 0.001 | 3.892 | 0.000^**^ |
| Sex | -0.023 | 0.018 | -1.267 | 0.205 | Sex | -0.022 | 0.019 | -1.198 | 0.231 |
| SBP | 0.000 | 0.001 | 0.169 | 0.866 | SBP | 0.000 | 0.001 | 0.176 | 0.860 |
| Triglycerides | -0.005 | 0.004 | -1.182 | 0.238 | Triglycerides | -0.005 | 0.004 | -1.107 | 0.269 |
| Total Cholesterol | -0.002 | 0.006 | -0.333 | 0.739 | Total Cholesterol | -0.002 | 0.006 | -0.344 | 0.731 |
| LDL-C | 0.003 | 0.011 | 0.243 | 0.808 | LDL | 0.003 | 0.011 | 0.285 | 0.776 |
| Glucose | -0.000 | 0.001 | -0.227 | 0.821 | Glucose | -0.000 | 0.001 | -0.185 | 0.853 |
| **SO** | 0.032 | 0.019 | 1.727 | 0.084 | **SO** | 0.032 | 0.019 | 1.742 | 0.082 |
| **AISI** | 0.013 | 0.012 | 1.058 | 0.290 | **SIRI** | 0.014 | 0.013 | 1.107 | 0.269 |
| **AISI*SO** | -0.001 | 0.018 | -0.051 | 0.959 | **SIRI*SO** | -0.008 | 0.018 | -0.430 | 0.667 |

Note: *P<0.05, **P<0.01; DHVD: Degenerative heart valve disease; SBP, systolic blood pressure; LDL-C, low-density lipoprotein cholesterol; SO: sarcopenic obesity; AISI, Aggregate Index of Systemic Inflammation; SIRI, Systemic Inflammatory Response Index
