# Supplementary material for: Association between sarcopenic obesity and cardiovascular diseases: the role of systemic inflammation indices
Source: Front Med (Lausanne). 2025 Jun 23;12:1581146. doi: 10.3389/fmed.2025.1581146 (PMC12230098; doi:10.3389/fmed.2025.1581146)
Supplement: Supplementary file 3 [file Table_3.docx]

**Table S3.** Moderating effect of AISI and SIRI on the relationship between SO and carotid atherosclerosis

|  | AISI | | | |  | SIRI | | | |
| --- | --- | --- | --- | --- | --- | --- | --- | --- | --- |
|  | β | SE | t | p |  | β | SE | t | p |
| Constant | -0.287 | 0.129 | -2.224 | 0.026^*^ | Constant | -0.286 | 0.129 | -2.216 | 0.027^*^ |
| Age | 0.004 | 0.002 | 2.324 | 0.020^*^ | Age | 0.004 | 0.002 | 2.280 | 0.023^*^ |
| Sex | -0.041 | 0.026 | -1.604 | 0.109 | Sex | -0.038 | 0.026 | -1.464 | 0.143 |
| Systolic BP | 0.001 | 0.001 | 1.710 | 0.088 | Systolic BP | 0.001 | 0.001 | 1.706 | 0.088 |
| Triglycerides | 0.004 | 0.006 | 0.624 | 0.533 | Triglycerides | 0.004 | 0.006 | 0.622 | 0.534 |
| Total Cholesterol | 0.015 | 0.008 | 1.958 | 0.051 | Total Cholesterol | 0.015 | 0.008 | 1.966 | 0.050^*^ |
| LDL | -0.002 | 0.015 | -0.118 | 0.906 | LDL | -0.001 | 0.015 | -0.084 | 0.933 |
| Glucose | -0.000 | 0.001 | -0.520 | 0.603 | Glucose | -0.000 | 0.001 | -0.546 | 0.585 |
| **SO** | 0.048 | 0.026 | 1.870 | 0.062 | **SO** | 0.048 | 0.026 | 1.856 | 0.064 |
| **AISI** | 0.016 | 0.017 | 0.928 | 0.354 | **SIRI** | 0.012 | 0.017 | 0.705 | 0.481 |
| **AISI*SO** | -0.010 | 0.025 | -0.423 | 0.672 | **SIRI*SO** | -0.002 | 0.025 | -0.084 | 0.933 |

Note: *P<0.05, **P<0.01; SBP, systolic blood pressure; LDL-C, low-density lipoprotein cholesterol; SO: sarcopenic obesity; AISI, Aggregate Index of Systemic Inflammation; SIRI, Systemic Inflammatory Response Index
